# Application of Information Theory to Computer Vision and Image Processing II

**DOI:** 10.3390/e28050486

**Published:** 2026-04-23

**Authors:** Wendy Flores-Fuentes, Oleg Sergiyenko, Julio C. Rodríguez-Quiñonez, Jesús E. Miranda-Vega

**Affiliations:** 1 Facultad de Ingeniería, Universidad Autónoma de Baja California, Mexicali 21280, Mexico; julio.rodriguez81@uabc.edu.mx; 2 Instituto de Ingeniería, Universidad Autónoma de Baja California, Mexicali 21100, Mexico; srgnk@uabc.edu.mx; 3 Tecnológico Nacional de México, IT de Mexicali, Mexicali 21376, Mexico; elias.miranda@itmexicali.edu.mx

Information theory is the foundation for computer vision and image processing across diverse applications. It constitutes the application of sophisticated mathematical principles to electrical engineering development for advanced research in vision systems and machine learning.

Within this field of study, 3D visual applications interact with three-dimensional models for various purposes, utilizing processes that emulate human behavior. These applications require hardware and software, supported by a framework that enables the acquisition of data on physical conditions, their codification, processing, and decoding, and data transmission for actuation. Point clouds are foundational in many applications; however, such applications aim for high-resolution point clouds, which are complex, high-dimensional and resource-intensive and have a fidelity bias. This Special Issue addresses point cloud acquisition and processing error management, point cloud completion and segmentation, and reconstruction techniques. Processing point cloud methods suffer from issues related to rough localized structures, which exhibit abrupt changes in geometry, such as peaks, gaps, and irregular edges. Furthermore, point clouds from both scans and photogrammetry contain noise and points that are not uniformly distributed, resulting in low or irregular density and thus causing a loss of precision. Most existing geometric-based methods assume non-rough surfaces to apply functions with smooth approximations, leading to a loss of detail. However, deep learning-based methods for point clouds have gained significant traction among researchers for their ability to capture the overall distribution of an object’s point cloud. Nevertheless, some methods present a lack of attention to local details in the global optimization, struggling to preserve intricate local details within point clouds. Therefore, segmenting the point cloud according to semantic parts can effectively mitigate these challenges via multi-modal point cloud assignment modules that allocate point clouds to different local parts incorporating textual embeddings. This enables deep learning networks to learn local geometric features and produce results with clearly defined local structures [1,2,3,4,5]. Other techniques involve noise reduction in the point clouds, the detection and elimination of outliers, entropy reduction, and smoothing, the results of which can be evaluated by point cloud registration algorithms such as iterative closest point and random sample consensus registration to compare the original and processed 3D point clouds [6,7]. It can be concluded that scanned data often exist in rudimentary forms, such as point clouds or meshes, which lack direct expressions of structure and semantic information inherent to objects. On the contrary, the automated generation of geometric models from point cloud data holds significant importance in the field of computer vision and has expansive applications, such as shape modeling and object recognition. Deep learning networks with recursive PointConv operations on nested subdivisions of point sets effectively extract features, segment point clouds, and accurately identify and compute parameters of regular geometric primitives with notable resilience to noise. The importance of this data enhancement process has been emphasized in the fields of constructive solid geometry and optical sensor fusion, which contribute to 3D reconstruction. This improves the precision of point cloud segmentation, geometric primitive parameter estimation, and our understanding of complex 3D structures [8,9,10,11].

Moving beyond 3D geometric representations, similar challenges pertaining to data acquisition, redundancy, and efficient representation also arise in image processing and compression. The discussion now turns to image processing and data compression theory for many computer vision tasks that require image super-resolution, de-noising, and image registration for applications such as medical imaging, remote sensing, and infrastructure health diagnosis. Compressed sensing (CS) is a popular data compression theory, enabling sub-Nyquist sampling, data compression, and flexible measurement rates for further reconstruction; however, when applied to images, it is hindered by high reconstruction complexity due to the high computational time needed for sparse estimation. This necessitates the incorporation of deep learning methods into CS estimation via two approaches. The first uses a convolutional neural network (CNN) to set up end-to-end models from the CS measurement data to the final image. The second builds a neural network module for each iteration step in the traditional optimization-based methods. Deep learning-based methods are not as sensitive to iteration steps as optimization methods are, but they are more sensitive to the sparse basis and measurement matrix and improve the reconstruction speed [12,13]. Since the core problem of CS reconstruction is using the measurement matrix and sparse basis to determine weightings or “sparse coefficients”, a deep learning model can be used to predict them; this renders the reconstruction results much more controllable as each weighting has a clear physical meaning. Integrating the CS mechanism into the network to prevent error propagation successfully reconstructs extremely low measurement rates that are impossible for traditional optimization-theory-based methods to achieve [14,15]. These data efficiency and reconstruction challenges become even more pronounced when dealing with high-dimensional imaging modalities. Hyperspectral images (HIS) are immense, collected by hundreds of contiguous spectral bands, where the size of each HSI can easily reach hundreds of megabytes. This can engender logistical problems in terms of storage, transmission, and processing. Lossless and near-lossless compression algorithms can be used for HISs. Lossless compression allows for the exact reconstruction of the image at the expense of realizing modest compression ratios, whereas near-lossless compression achieves higher compression ratios while limiting the pixel distortion to a pre-defined absolute or relative error [16]. Beyond compression and transmission, another fundamental problem in image processing concerns the recovery of lost or degraded spatial information. Despite the diversity of proposed approaches, the core challenge in image super-resolution lies in balancing perceptual realism with faithful structural reconstruction. Regarding image super-resolution, the goal is to recover high-frequency information that is missing from low-resolution images, a task that poses significant challenges. Traditional methods are constrained by algorithmic resolution limits and unrealistic detail recovery, making them inadequate for practical applications such as remote sensing, surveillance and identity recognition. In contrast, methods based on deep learning, such as CNNs and generative adversarial networks (GANs), improve reconstruction quality, although the generated textures are overly smooth and lack sufficient high-frequency details if the network depth and introduced mechanisms such as residual learning are inadequate. Two major issues arise in complex scenarios: models are more prone to inconsistent local texture reconstruction due to a lack of semantic understanding, and it it is hard to improve texture generation and reduce artifacts. This Special Issue addresses this by introducing semantic priors, which enhance the model´s understanding of image structure while abstracting convolutionally extracted texture information into feature representations. The texture data from previous layers are treated as feature vertices, with their relationships represented by an adjacency matrix, enabling contextual dependencies. This allows the model to capture long-range contextual relationships, effectively utilizing semantic information to reduce semantic inconsistencies in reconstructed images [17,18,19]. In addition to resolution limitations, imaging conditions themselves often impose severe constraints on visual quality. Although numerous enhancement techniques have been proposed, low-light image processing remains fundamentally constrained by noise amplification and color distortion. Another challenge lies in enhancing the visual quality of images taken under complex low-light conditions, which is crucial for effectively improving the performance of methods such as object detection and scene understanding at night or in low-light conditions. Over the past few decades, methods have typically been predicated on hand-designed features and processing steps such as histogram equalization and gamma transformation. These methods are simple and fast, but they usually amplify noise while enhancing the image and often cannot restore the color and details of low-light images well. End-to-end networks based on deep learning usually utilize pixel-level error functions to optimize models and have difficulty effectively modeling real visual errors between the enhanced and normally exposed images. One solution is to employ an adaptive dual aggregation encoder, built to fully explore the global properties and local details of low-light images to extract illumination-robust features. Then, a reversible normalizing flow decoder is used to model real visual errors between enhanced and normally exposed images by mapping images into underlying data distributions. To further improve the quality of the enhanced images, a gated multi-scale information transmitting module is utilized to introduce multi-scale information from the adaptive dual aggregation encoder into the normalizing flow decoder, reconstructing brighter, more detailed images from low-light images [20]. When illumination conditions deteriorate even further, image degradation extends beyond low visibility to severe structural and textural loss. In extremely dark conditions, images taken in ultra-micro-light environments usually have inherent defects such as very low brightness and contrast, a high noise level, and a serious loss of scene details and colors, presenting numerous challenges in object detection and classification. However, experiments using image preprocessing methods have been conducted, utilizing the contrast limited adaptive histogram equalization (CLAHE) algorithm and gray-level co-occurrence matrix (GLCM) to extract texture features of the enhanced images. These features are then used as input to a backpropagation neural network classification model, performing efficiently [21,22]. Such enhancement strategies are particularly important for downstream high-level vision tasks that rely on fine structural cues. These challenges directly impact scene understanding tasks that depend on precise edge and contour information. For instance, in arbitrary shape scene text detection, lighting can affect the results. Regarding scene text detection, segmentation-based methods mostly rely on complicated post-processing steps, and the post-processing operation is separate from the training process, which greatly reduces detection performance. Under this consideration, a network employing the binarization of hyperbolic tangents optimizes the converge speed by reducing the number of deep learning epochs. This is due the fact that features from different channels in the same-scale feature map focus on information derived from different regions in the image to better represent the important features of all objects in the image [23,24,25]. Related challenges also emerge in industrial vision applications, where geometric distortions further complicate text and symbol interpretation. Another application of text detection is instrument reading in industrial scenarios, which poses significant challenges due to reading contour distortion caused by perspective transformation in the instrument images. This is caused by the incorrect labeling of target box vertices, which arises from the vertex entanglement problem; this can be addressed by a quadrilateral contour disentangled detection network based on a multi-scale feature pyramid network for effective feature extraction. Additionally, a polar coordinate decoupling representation can be used to model each side of the instrument contour using polar coordinates [26,27,28,29,30]. In almost all applications, severe occlusion and adhesion exist among multiple targets, making it difficult to accurately distinguish between them. CNNs have dominated semantic segmentation; however, they have several drawbacks, such as limited receptive fields and the inability to capture global information, which significantly reduces their segmentation accuracy under severe occlusion. By contrast, Transformers exhibit stronger performance and flexibility compared to CNNs. Transformer-based visual recognition networks offer better computational efficiency and feature extraction capabilities when optimized with a feature pyramid network (FPN) to replace the original MLP layer, enhancing the semantic information of features [31,32]. While segmentation focuses on spatial understanding within single images, many real-world applications require modeling temporal dynamics. On the other side, computer vision and image processing involve video processing, where human activity recognition plays a crucial role by learning spatiotemporal representations from motion pictures. Deep learning networks that employ channel and spatial attention mechanisms emphasize key features and suppress irrelevant ones, effectively capturing key behavioral features in videos using both attention mechanisms [33,34,35]. Beyond purely visual representations, recent advances increasingly highlight the integration of visual and linguistic information. This leads us to explore the incorporation of natural language processing into computer vision through multimodal learning. Transformer models have been proven to be more effective in capturing long-range dependencies in data and achieving superior performance compared to CNNs and long short-term memory (LSTM) networks. Image captioning is important for improving the intelligence of systems; its main task is to understand the context of the image and interpret it with natural language to realize the mutual mapping between image and text features. This necessitates the construction of a label text description dataset and a Swing Transformer to extract semantic and stylistic features from images using encoders. A decoder integrates the image information into text features, and via an interaction between the image information and text features, content-appropriate sentences are generated word by word. Finally, sentence style loss is added to the total loss function to render the style of generated sentences closer to the training set [36]. Recent advancements in large pre-trained vision–language models have been notably successful, with contrastive language–image pre-training (CLIP) standing out for its ability to model the relevance between images and text. This model enhances understanding by aligning visual content with textual descriptions. This strategy effectively bridges the domain gap between visual and linguistic features, enabling comprehensive target activation for accurate pixel-level labeling [37,38,39].

This Special Issue aimed to explore information theory, measurement methods, data processing, tools, and techniques for the design and instrumentation used in machine vision systems using the application of computer vision and image processing. It brought together theoretical foundations and practical methodologies to analyze, process, and understand visual data through unified perspectives on information content, redundancy, and statistical structure.
